# BRAF p.V600E-Negative Langerhans Cell Histiocytosis Associated with a Periapical Cyst: A Case Presentation with Broad Review of the Differential Diagnosis and Disease Pathophysiology

**DOI:** 10.1007/s12105-025-01763-5

**Published:** 2025-03-15

**Authors:** Anneze Odendaal, Ashwin Kassan, Leon Janse van Rensburg, Amir H. Afrogheh

**Affiliations:** 1 Department of Oral Medicine and Periodontology, Faculty of Dentistry, University of the Western Cape, Cape Town, South Africa; 2 Specilaist, Netcare Greenacres Hospital, Port Elizabeth, South Africa; 3 Division of Radiodiagnosis, Department of Medical Imaging and Clinical Oncology, Faculty of Medicine and Health Sciences, Stellenbosch University, Cape Town, South Africa; 4 Department of Oral and Maxillofacial Radiology, Faculty of Dentistry, University of the Western Cape, Cape Town, South Africa; 5 Department of Oral and Maxillofacial Pathology, Faculty of Dentistry, University of the Western Cape and National Health Laboratory Service, Tygerberg Hospital, Cape Town, South Africa; 6 Division of Anatomical Pathology, Faculty of Medicine and Health Sciences, Stellenbosch University, Cape Town, South Africa

**Keywords:** Periapical (Radicular) cyst, Langerhans cell histiocytosis, BRAF, Langerin (CD 207), CD1a

## Abstract

**Background:**

Langerhans cell histiocytosis (LCH) rarely presents in the oral and maxillofacial region, and while isolated and small collections of Langerhans-type cells have been found in periapical cysts, there have been no reported cases of LCH arising in periapical cysts.

**Methods:**

A 58-year-old female presented with isolated erythematous dry skin lesions and a radiolucent lesion of the anterior maxilla. Microscopic examination of the enucleation specimen revealed a periapical cyst with large collections of atypical cells with grooved folded nuclei with eosinophils consistent with LCH. Immunohistochemistry (IHC) was performed to confirm the diagnosis. *BRAF* mutation status was evaluated with the *BRAF* p. V600E antibody and the automated real-time PCR-based Idylla™ assay, capable of qualitative detection of 5 mutations in codon 600 of the *BRAF* gene.

**Results:**

The LCH cells were positive for S100, CD1a, and Langerin (CD 207) and negative for *BRAF* p. V600E mutations. Ki-67 was 45%.

**Conclusion:**

The association of LCH with a periapical cyst could be explained by the active surveillance and migration of neoplastic Langerhans-type cells in blood to the site of apical chronic inflammation, in a patient with LCH. Careful attention to morphologic features in conjunction with Langerin IHC, helps exclude other closely-related dendritic tumours. *BRAF* p. V600E testing, ideally with real-time PCR assays, can help identify patients who may benefit from *BRAF* inhibitor therapies. New generations of sequencing that cover a large panel of genetic alterations beyond the frequent *BRAF* p. V600E mutations (e.g. rare in-frame *BRAF* deletions), could provide valuable information about the extent, prognosis and treatment of LCH patients.

## Introduction

Langerhans cell histiocytosis (LCH) is a disease characterized by an abnormal proliferation of Langerhans-type cells [1]. Langerhans cells (LC) are unique granule-containing dendritic cells that are primarily found in the epidermis [2]. LC are able to internalize antigens and migrate through lymphatic channels into the regional lymph nodes, where they differentiate into interdigitating dendritic cells [3]. Interdigitating dendritic cells present the processed antigen to T lymphocytes. Activated T lymphocytes then stimulate differentiation of B lymphocytes into plasma cells with subsequent release of immunoglobulins.

The prevailing notion is that LCH is a truly neoplastic process. The clonal nature of the LCH cells and the discovery of *BRAF* genetic alterations support a neoplastic condition [4]. 

Pringle et al. described a series of six cases of chronic apical disease with small collections of Langerhans-type cells and speculated that such cases may represent early or mild forms of LCH [5].

In the present article, we report a unique case of LCH associated with a periapical cyst in an adult patient with multisystem disease. In addition, this report provides an overview of the current state of knowledge on etiology, head and neck manifestations, diagnosis and management of LCH.

## Case Report

### Clinical and Radiological Presentation

**Fig. 1:**
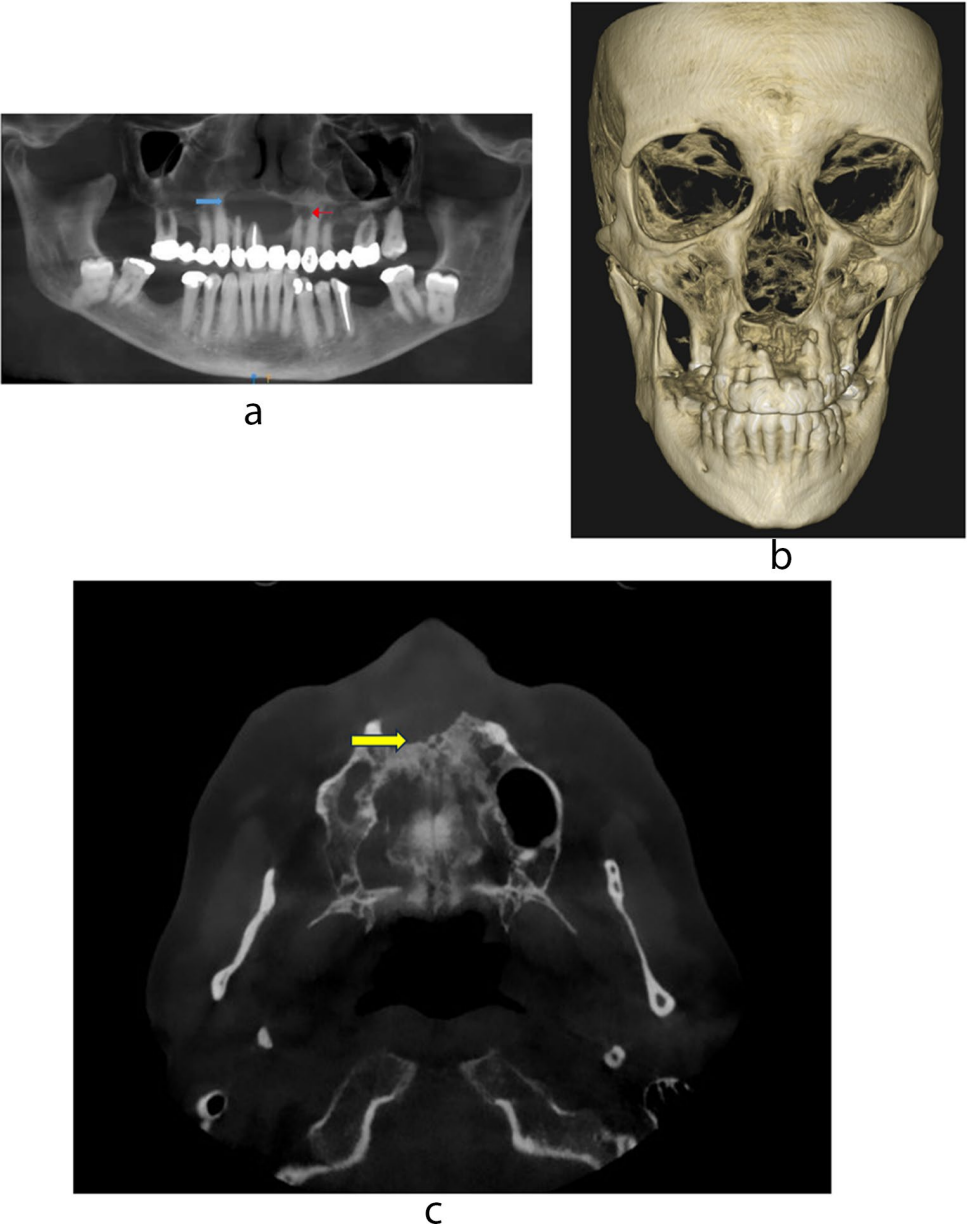
**a** CBCT panoramic reconstruction shows a large well circumscribed multilocular radiolucency associated with the apices of teeth 10,9 and 7. Root canal treated tooth 9 suggests a periapical cyst (blue arrow larger locule and red arrow smaller locule). **b** A 3D MIP shows destruction of the anterior maxillary bone. **c** CBCT scan shows the posterior border of the lesion with cortical bone destruction and narrow transition zone involving nasopalatine foramen (yellow arrow)

A 58 year-old Caucasian female was referred by her dentist to an oral and maxillofacial surgeon for a non-resolving abscess associated with the apex of a root canal-treated right central maxillary incisor (tooth 9, Universal Tooth Numbering System). The patient had an equestrian incident several years prior, that resulted in fall and consequent anterior dental trauma. Dry and isolated erythematous skin lesions on the patient’s arms and legs were noted on extra-oral examination. Intraoral examination revealed extensive maxillary bridge work. The four-unit anterior maxillary bridge (extending from tooth 10 to 7) was mobile with purulent discharge from the tender right upper buccal sulcus. A reconstructed panoramic radiograph (PAN) from a Cone Beam Computed Tomography (CBCT) scan showed an apparently well circumscribed large multilocular radiolucency extending from tooth 11 to 7 (Fig. 1a). The larger compartment extended from tooth 11 to 6 with destruction of the right hard palate (Fig. 1a, blue arrow), and the smaller well-defined compartment was apical to tooth 7 (Fig. 1a, red arrow). The 3D MIP (Maximum Intensity Projection) showed destruction of the anterior maxillary bone (Fig. 1b). The posterior border of the lesion showed a pattern of sharp bone destruction and narrow transition zone involving the nasopalatine foramen. The anterior foramen of Scarpa was completely destroyed and the right foramen of Stenson was partially eroded (Fig. 1c, yellow arrow).

The panoramic radiological differential diagnosis, given a background of extensive dental disease with chronic infective/inflammatory pathology and previous trauma, included a solitary or multiple periapical cyst(s), odontogenic keratocyst, ameloblastoma and chronic osteomyelitis.

The aggressive pattern of bone destruction on CBCT raised a differential diagnosis of a primary or metastatic malignant disease such as squamous cell carcinoma, lymphoma, multiple myeloma, Brown tumor of hyperparathyroidism and granulomatous infections such as tuberculosis.

Cortical destruction and a narrow transition zone is a common radiologic finding, and not very useful in distinguishing between malignant and benign lesions. Complete destruction may be seen in high-grade malignant lesions, but is also seen in locally aggressive benign lesions like LCH and osteomyelitis, especially in patients over 40 years of age.

The clinical diagnosis was that of a periapical cyst. Teeth 10,9 and 7 were extracted and pontic 8 was removed. The cystic lesion was enucleated under general anaesthesia. The tissue was placed in a container containing formalin and submitted for histopathologic evaluation. The postoperative plan included: (1) fitting of an immediate removable maxillary partial denture (2) an iliac crest bone graft due to significant bone loss and (3) rehabilitation using dental implants or a fixed partial prosthesis.

### Histologic Features

**Fig. 2:**
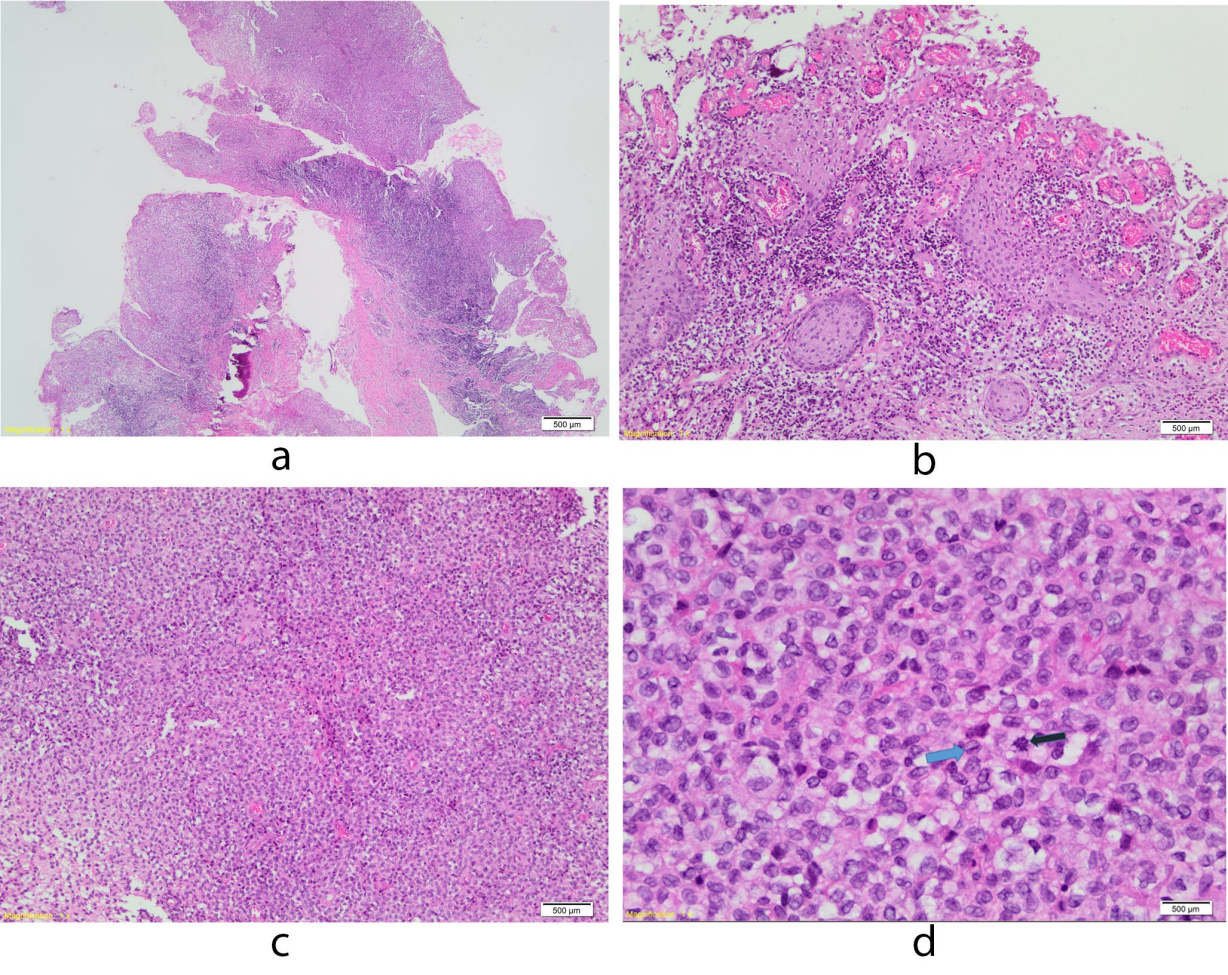
(**a**) The photomicrograph shows an inflammatory odontogenic cyst (H&E, x 4). (**b**) The cyst is lined by a proliferative squamous epithelium with spongiosis. Transmigration of neutrophils into the lining epithelium is clearly seen with chronic inflammation of the cyst’s connective tissue capsule (H&E, x 10). (**c**) The image shows a large solid population of atypical eosinophilic cells (H&E, x 20). (**d**) Close microscopic examination reveals atypical cells with grooved folded nuclei (coffee bean-shaped nuclei) with scattered eosinophils and atypical mitoses (blue arrow, a coffee bean-shaped nucleus and black arrow an atypical mitotic figure) (H&E, x40)

Histologic examination of the enucleation specimen revealed a periapical (radicular) cyst lined by a hyperplastic and spongiotic stratified squamous epithelium with intraepithelial neutrophils (Fig. 2a and b). The fibrous connective tissue capsule of the cyst showed chronic inflammation and a large focus with a dense population of atypical histiocytes with grooved and folded nuclei (coffee bean-shaped nuclei) (Fig. 2c). There were scattered atypical mitotic figures and eosinophils (Fig. 2d).

## Methodology

### Immunohistochemistry

Immunohistochemistry (IHC) was performed on 5-µm formalin-fixed paraffin-embedded (FFPE) sections from the tissue block. Slides were stained with a *BRAF* p.V600E-directed antibody (clone VE1, dilution 1:200, pH9, Eurobio), S100, CD1a, Langerin (CD 207) and Ki-67. As a negative control, primary antibody was omitted. Appropriate positive external controls were included on each stained slide. The stains were analyzed by one pathologist (AA) blinded to molecular results. The *BRAF* immunostain was scored as follows: positive (with the percentage of VE1 + LCH cells) when viable LCH cells harbored cytoplasmic staining, negative when no staining or only scarce VE1 + LCH cells or only VE1 + macrophages.^6^

### Detection of BRAF exon 15 Mutations with the Idylla™ Molecular Diagnostic System

Briefly, a tumor-rich portion of tissue was macrodissected from glass slides, or an FFPE curl/scroll was inserted into the Idylla cartridge. The Idylla™ system (Biocartis, Mechelen, Belgium) is a fully automated real-time PCR-based system for molecular diagnostics. Single-use cartridges contain all the necessary reagents to perform sample lysis, DNA extraction and real-time PCR amplification. The Idylla console software analyses the fluorescence signals and reports the presence or absence of a mutation. The Idylla Assay cartridges are designed to detect five mutations in codons 600 of the *BRAF* gene [6].

## Results

**Fig. 3:**
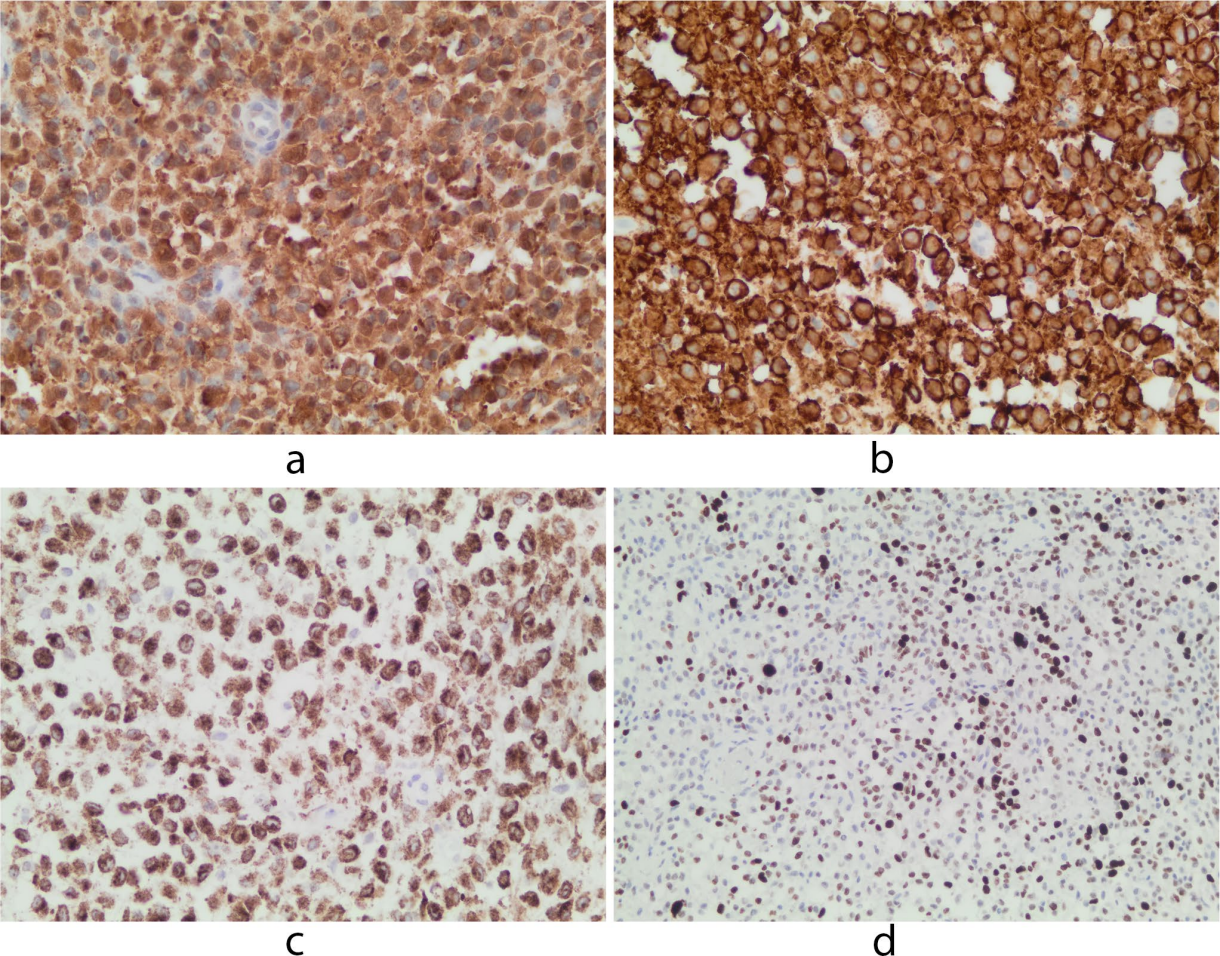
(**a**) The photomicrograph shows nuclear and cytoplasmic staining of LCH cells for S100 protein (IHC, x 40). (**b**) Strong membranous staining for CD1a (IHC, x 40). (**c**) Intense granular cytoplasmic positivity for Langerin (CD207) (IHC, x 40). (**d**) The proliferation index, Ki-67, is 45% (IHC, x 20)

The atypical histiocytes showed marked nuclear and cytoplasmic staining for S100 protein (Fig. 3a), intense membranous staining for CD1a (Fig. 3b) and strong granular cytoplasmic positivity for Langerin (CD207) (Fig. 3c). CD68 was positive in macrophages. The Ki-67 proliferative index was 45% (Fig. 3d).

IHC and the Idylla™ PCR-based assay were both negative for *BRAF* p.V600E.

Based on the microscopic features and the immunohistochemical profile, a diagnosis of *BRAF* p.V600E-negative LCH arising in the background of a periapical cyst was established. In addition, the presence of the skin lesions and elevated Ki-67 proliferation index (> 40%) were suggestive of multisystem LCH. The patient was referred to an oncologist for a complete work up (skeletal survey, skull series, bone scan, complete blood count, sedimentation rate, liver function tests, electrolytes and urinalysis) and management. Unfortunately, the patient failed to keep her scheduled oncology clinic appointment and was subsequently lost to follow up.

## Discussion

In 1868, Paul Langerhans, as a medical student, described an epidermal cell population with characteristic dendrites using a novel “gold colloid” stain. Langerhans referred to these cells as extra-cutaneous nerves [7]. Over a century later and with the advent of electron microscopy, Langerhans’ neural doctrine was challenged and it was shown that LC are a specific subset of dendritic cells that play a pivotal role in immunity [8]. The ultrastructural hallmark of epidermal LC is the presence of cytoplasmic pentalaminar membranous granules, termed Birbeck granules. The Birbeck granules have a tennis-racket shape and are 200–400 nm long and 33 nm wide, with a zipper-like appearance [9]. The granules originate from cellular membranous structures that are internalized during the process of endocytosis [8]. Epidermal LC express Langerin (CD207), a lectin needed for the production of Birbeck granules [10]. More recently, the immunoexpression of Langerin (CD 207) has mainly replaced transmission electron microscopy for identification of LC.

LCH is defined by World Health Organization (WHO) as clonal neoplastic proliferation of Langerhans-type cells [11]. LCH is a rare disease. Over 50% of the LCH cases are diagnosed in patients under 15 years of age. LCH is extremely rare in adults (1 case per million per annum) [12]. Clinically, LCH is classified according to the number of involved organs. Systemic involvement is classified as unisystemic or single system (involvement of a single organ) or multisystemic (two or more organs involved); within each organ the disease is categorized as unifocal or multifocal (one or more than one lesion, respectively) [12]. Bones are frequently affected, followed by the skin. Head and neck manifestations of LCH are frequent, with reported frequencies varying from 50–80%.^13^ In a large case series, 54% of patients had craniofacial osseous involvement, which included the calvaria, skull base, temporal, and maxillofacial bones [13]. Following calvaria and usually affecting 10% of LCH patients, the mandible is the second most common bone involved in LCH [13]. The posterior region is the most common location for mandibular lesions [12]. The gingival tissues are frequently affected in patients with gnathic LCH, with reported frequencies of up to 20%.^13^ LCH involving only soft tissues may also occur, particularly the gingiva and hard palate [12].

Radiologically, LCH may present as a unilocular or multilocular radiolucency (Fig. 1a) with bone loss and floating teeth [14]. Periapical presentations of LCH are common [14, 15]. In a study of 118 cases of non-endodontic periapical lesions, squamous cell carcinoma was the most predominant malignancy misdiagnosed as apical periodontitis on radiological examination (*n* = 7, 5.93%), followed by adenoid cystic carcinoma (*n* = 1, 0.85%), and LCH (*n* = 1, 0.85%) [16].

Subtle radiological signs of an aggressive lesion in an apparently benign appearing radiolucency should alert to the possibility of a locally aggressive or malignant lesion.

Cases with multiple punched out lytic lesions of the skull closely resemble multiple myeloma [17]. Skin lesions are characterized by erythematous eruptions or a discreet pinkish-brown papule [18].

Histologically, a solid infiltrate of cells with grooved folded nuclei (coffee bean-shaped nuclei) is seen in LCH. The reactive inflammatory component consists of eosinophils. In addition, neutrophils, lymphocytes, macrophages, plasma cells, and multinucleated giant cells may also be seen [19]. Atypical mitoses and apoptotic bodies have also been described, with necrosis observed in cases with a high proliferation index [19]. In one study, a Ki-67 proliferative index of > 40% was correlated with the presence of multisystem disease [19].

LCH shares surface markers with epidermal LC (CD1a+/CD207+).^4^ Histomorphological and immunohistochemical similarities between LCH cells and epidermal LC have resulted in the persistent view that LCH is a disorder of transformed or activated epidermal LC. More recently, dendritic-cell subsets have been discovered with the potential to express Langerin (CD 207) and form Birbeck granules, that survey tissues with increased recruitment from blood to tissue during inflammation [20]. Badalian-Very and colleagues have detected *BRAF* p.V600E in 57% of LCH lesions [21]. In addition to *BRAF* p.V600E, other activating mutations in *BRAF*, including in-frame deletions, fusions, and duplications, have been identified in LCH lesions [4]. Thus, LCH cells are more likely to arise from dysregulated differentiation of bone marrow–derived precursor cells with activating MAPK somatic mutations rather than from transformed or activated epidermal LC [4]. The latter is known as the “misguided-myeloid-differentiation” hypothesis [4]. Similarly, we hypothesize that, the chronic inflammatory microenvironment in a periapical cyst encourages active recruitment of genetically altered Langerhans-type cells from blood to the connective tissue capsule of the cyst, in a patient with established LCH.

A deeper insight into the evolution and differentiation of dendritic cells, may help better understand the differential diagnosis and immunoexpression of LCH.

Monocytes characteristically express CD163, S100, and OCT2 [22]. As they migrate into tissues and assume a macrophage phenotype, they exhibit enhanced expression of CD163 and Factor XIIIA with low expression of S100 and OCT2 [22]. Alternatively, if monocytes differentiate toward a dendritic cell phenotype, they show decreased expression of CD163 and OCT2. Indeterminate dendritic cells that are presumed to be LC precursors, express S100 protein, CD1a, and CD4, but are negative for Langerin (CD 207); interdigitating dendritic cells express S100 protein and CD4, but are negative for CD1a and Langerin (CD 207) [22]. Differentiation toward a mature LC phenotype is demonstrated by expression of CD1a and Langerin (CD 207) [22]. Therefore, the histological differential diagnosis of LCH includes granulomatous inflammatory disease (e.g. tuberculosis or fungal infections) and neoplasms in the same line of differentiation as LCH, namely, Langerhans cell sarcoma (LCS), interdigitating dendritic cell sarcoma, and indeterminate dendritic cell tumor (IDCT).

Coffee bean-shaped nuclei are not specific to LCH and may be seen in the setting of inflammatory conditions such as granulomatous inflammation. Careful attention to the arrangement of cellular components (granulomas) and IHC helps exclude granulomatous inflammatory disease. Distinction from LCS and IDCT may be challenging as both lesions show CD1a and S100 expression [22]. In addition *BRAF* p.V600E has been reported in LCS and IDCT [23].

In contrast to LCH, LCS is an aggressive malignancy, in which the Langerhans-type cells possess high-grade large and markedly pleomorphic round to oval vesicular nuclei with clumped chromatin and prominent nucleoli. Focally, some cells may exhibit the complex grooves of LCH cells, a clue to diagnosis, however, in general the diagnosis of LCS is challenging and often requires an extensive panel of immunohistochemical markers to exclude tumors with high-grade nuclei, including carcinoma, melanoma, lymphoma, and sarcoma [3, 11, 24]. The immunophenotype of LCS is identical to that of LCH, positive for CD1a and Langerin (CD 207) [3, 11, 24]. The percentage cut-off in the expression of CD1a and Langerin (CD 207) to establish a diagnosis of LCS is not defined by the WHO. One study has suggested strong CD1a and Langerin (CD 207) expression in more than 30% of LCS cells [22]. LCS shows variable eosinophil infiltrates and high mitotic activity, with more than 50 mitoses per 10 high-power fields [11].

IDCT is defined by the WHO as neoplastic proliferation of spindled to ovoid normal indeterminate cells, the presumed precursor cells of LC [25]. The IDCT cells resemble LCH cells with irregular nuclear grooves and ample eosinophilic cytoplasm and express dendritic cell markers, CD1a and S100. However, they lack the Birbeck granules of LCH on ultrastructural examination and do not express Langerin (CD 207) [3, 25]. The mitotic rate is variable from case to case and there is usually a mixed inflammatory cell infiltrate consisting of eosinophils, small reactive lymphocytes, macrophages, and multinucleated giant cells. IDCT are negative for the histiocytic marker, CD163 and follicular dendritic cell markers CD21, CD23 and CD35 [3, 25].

Interdigitating dendritic cell sarcoma is characterized by the proliferation of spindled to ovoid cells showing interdigitating dendritic cell phenotype [3, 26]. Unlike LCH, solitary lymph node involvement is the most common clinical presentation, however, extra-nodal involvement (skin and soft tissue) have been reported [26]. The tumor cells display abundant eosinophilic cytoplasm, indistinct cellular borders, and spindled to ovoid indented nuclei with small conspicuous nucleoli. Mitotic activity is low, with less than 5 mitoses per 2 mm [2] and Ki-67 proliferative index of 10–20%.^26^ Multinucleated tumor cells are occasionally seen. Different from LCH, interdigitating dendritic cell sarcoma grows in sheets, fascicles and whorls. Small lymphocytes are interspersed throughout the tumor. The tumor cells consistently express S100 protein and vimentin and are negative for CD1a, Langerin (CD 207), CD21, CD23 and CD35 [11, 22].

*BRAF *IHC, using the monoclonal antibody VE1, has been validated to recognize a mutant *BRAF* p.V600E protein with a negative predictive value of 92%.^6^*BRAF* IHC is efficient for detecting the V600E genetic variant, but negative cases should be further evaluated by DNA-based techniques for other *BRAF* variants, which have also been shown to drive clinical benefit from *BRAF* inhibitors [6]. DNA-based real-time PCR based assays are widely available in many laboratories across the world, including pathology laboratories in developing countries. One such assay is the automated Idylla™ assay with a positive- and negative predictive value of 100% for *BRAF* p.V600E [6, 27]. While we failed to demonstrate a somatic *BRAF* p.V600E variant by IHC and PCR-based assay, the possibility of other activating mutations in *BRAF*, especially in-frame deletions cannot be entirely excluded. In fact, in-frame *BRAF* deletions are usually seen in patients with multisystem LCH compared to single system LCH, and especially in those with liver involvement with associated poor outcome [28]. *BRAF* deletions are resistant to “first-generation” *BRAF* inhibitors and are sensitive to mitogen-activated protein kinase kinase (MEK) inhibitors [28]. Thus, determination of *BRAF* deletions in LCH patients lacking *BRAF* p.V600E may have prognostic and therapeutic implications. A study of 31 adult patients with LCH found that unifocal oral involvement is significantly associated with multisystem disease [29]. A multidisciplinary approach to patient management should be adopted with thorough clinical and radiological examination, hematological tests (full blood count, platelet count, coagulation tests and liver function tests) and urine biochemistry [30]. Focal jaw lesions are best managed by curettage [29]. Intralesional corticosteroid injections, often help with pain management and encourage healing. The affected mucosal regions require periodontal treatment, ranging from prophylaxis to root planning, depending on the patient’s oral hygiene status. Tooth extractions should be restricted to areas with significant bone loss with lack of periodontal support [29]. The prognosis is good in most cases and the estimated three-year overall survival for adult patients is 94.4%.^28^ Liver or spleen involvement and age older than 50 years indicate poor prognosis [28].

## Conclusion

We have conclusively demonstrated that LCH can occur in association with a periapical cyst. Pathologists should consider the possibility of LCH in chronic apical lesions with atypical clinical and radiologic features, as this can be the first manifestation of LCH in a patient with multi-system disease.

## Data Availability

No datasets were generated or analysed during the current study.
